# Inherent change in MammoSite applicator three-dimensional geometry over time

**DOI:** 10.1186/1748-717X-2-37

**Published:** 2007-09-24

**Authors:** Subhakar Mutyala, Walter Choi, Atif J Khan, Ravi Yaparpalvi, Alexandra J Stewart, Phillip M Devlin

**Affiliations:** 1 Department of Radiation Oncology, Montefiore Medical Center, Bronx NY 10467, USA; 2 Department of Radiation Oncology, Albert Einstein College of Medicine, Bronx, NY 10461, USA; 3 Radiotherapy Department, Royal Marsden Hospital, Sutton, England, UK; 4 Department of Radiation Oncology, Brigham and Women's Hospital, Boston, MA 02115, USA; 5 Department of Radiation Oncology, Dana-Farber Cancer Institute, Boston, MA 02115, USA

## Abstract

Accelerated partial breast irradiation is commonly done with the MammoSite applicator, which requires symmetry to treat the patient. This paper describes three cases that were asymmetric when initially placed and became symmetric over time, without manipulation.

## Background

Accelerated partial breast irradiation (APBI) with the MammoSite catheter is a new brachytherapy concept in breast conserving therapy for a subset of patients with early stage breast cancer [1,2]. The catheter consists of an inflatable balloon and a central channel for HDR brachytherapy. The initial experience [3] describes the ideal technique for the initial placement of the catheter, either at the time of lumpectomy or percutaneously under ultrasound guidance. As the initial Phase I trial describes, in order to deliver a homogenous dose to the tumor cavity, the balloon on the catheter should be inflated with saline to achieve a uniform spherical shape. Asymmetry of the applicator, poor placement, and intrinsic applicator abnormalities are all grounds for removal of the applicator. In this trial, a number of applicators were removed, with poor balloon conformance the most common reason for removal. We describe three separate cases where asymmetric applicators corrected themselves over time without any intervention, allowing for subsequent treatment.

## Case Presentation

### Case 1

The first patient is a 72 year-old female with an abnormality noted on a screening mammogram. A stereotactic core biopsy showed invasive ductal carcinoma. The patient subsequently had a lumpectomy and axillary node dissection, with pathology revealing a well-differentiated 9 mm invasive ductal carcinoma with no lympho-vascular space invasion. Surgical margins were negative and all lymph nodes removed on axillary dissection were negative for tumor. The patient was seen in our department and had a full history, physical, and pathology review. Based on her history and pathology, she was deemed a candidate for APBI with the MammoSite applicator and was placed in our institutional protocol.

**Figure 1:**
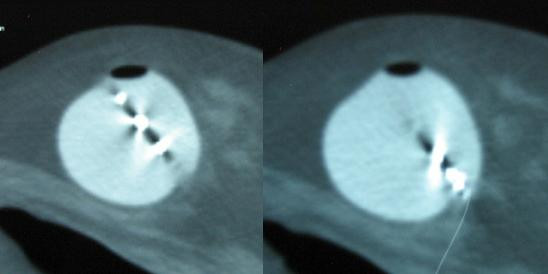
CT scan slices from case 1 showing the asymmetry of the center channel.

The patient returned for MammoSite placement by ultrasound guided percutaneous method approximately 6 weeks after her surgery. The MammoSite was placed successfully and inflated with 45 cc of contrast diluted with sterile water (1:10). Immediate CT scan for planning was performed with radio-opaque markers inserted into the isotope channel (figure 1). The scan revealed an asymmetrical applicator, with the isotope channel off center by 5 mm. The applicator was partially deflated, repositioned and re-inflated. The applicator was more symmetrical, yet still not ideal.

**Figure 2:**
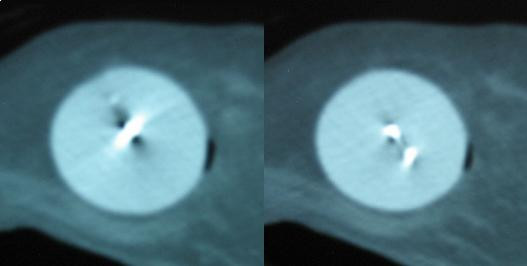
CT scan slices from case 1 showing the symmetry of the center channel.

The patient returned the following day. Under fluoroscopy (45 degree tangent with isocenter in center of applicator), it was noted the applicator had changed geometry from her initial film. The patient underwent a repeat CT scan using a radio-opaque marker in the isotope channel. The scan revealed an almost fully symmetrical sphere with regard to the isotope channel (figure 2). The patient was re-planned with a fully optimized custom plan, resulting in an acceptable dose distribution along the parameters of the protocol. The patient was subsequently treated to 34 Gy in 3.4 Gy BID fractions. The patient underwent a CT daily, confirming no further change in the applicator over the course of the treatment.

### Case 2

The second patient is a 75 year-old female with a density seen on a screening mammogram. A 6-month follow-up mammogram showed an interval increase in size while an MRI showed an enhancing area in the breast. A core biopsy was performed, showing poorly differentiated invasive ductal carcinoma with lobular features, ER+/PR+, with associated DCIS. The patient had a wire localized lumpectomy and sentinel node biopsy, with pathology revealing a 13 mm invasive ductal/lobular carcinoma, grade III, EIC negative, with no lympho-vascular space invasion. All surgical margins were negative for tumor and two sentinel nodes removed were negative for tumor. The patient requested accelerated partial breast irradiation with the MammoSite applicator. Based on her pathology and histology, she was deemed to be a suitable candidate for APBI.

**Figure 3:**
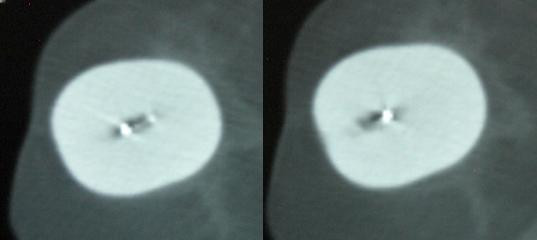
CT scan slices from case 2 showing the asymmetry of the center channel.

The patient returned for MammoSite placement by ultrasound guided percutaneous method approximately 4 weeks after her surgery. The MammoSite was placed successfully and inflated with 40 cc of contrast diluted with sterile water (1:10). A CT scan for planning was performed with radio-opaque markers inserted into the isotope channel (figure 3). The scan revealed an elliptical shaped applicator due to fibrous scarring.

**Figure 4:**
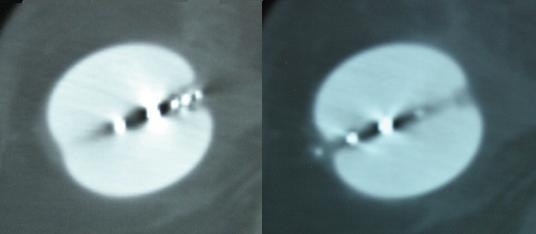
CT scan slices from case 2 showing the symmetry of the center channel.

The patient returned four days later (the following Monday). Under fluoroscopy it appeared the applicator had changed geometry. The patient was CT scanned again for re-planning, which revealed a perfectly symmetrical sphere (figure 4). The patient was re-planned using PLATO software with a fully optimized custom plan. The plan was acceptable and the patient was subsequently treated to 34 Gy in 3.4 Gy BID fractions. Again, the patient underwent a CT daily, which revealed no further change in the applicator geometry over the course of the treatment.

### Case 3

**Figure 5:**
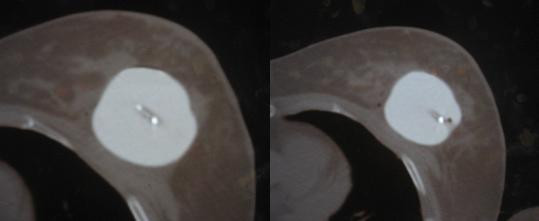
CT scan slices from case 3 showing the asymmetry of the balloon.

**Figure 6:**
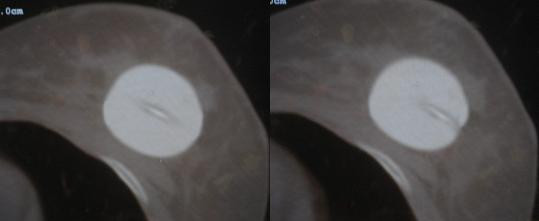
CT scan slices from case 3 showing balloon symmetry.

The third patient is a 61 year-old female who presented with a palpable mass in the upper inner quadrant of her left breast. Mammography revealed a 1 cm distortion of architecture at the 12 o'clock position of the left breast. Ultrasound-guided core biopsy revealed moderately differentiated infiltrating ductal carcinoma, which was ER/PR+ and HER-2/neu negative. She underwent breast-conserving surgery, with final pathology revealing a 2.5 cm tumor with negative margins of resection. Three sentinel lymph nodes were free of metastatic disease. After discussion of her treatment options, the patient elected to undergo APBI to complete her breast conserving therapy. Soon after consultation, she underwent percutaneous, ultrasonographically guided placement of the MammoSite device, which was inflated with 45 cc of 10% hypaque solution. The planning CT scan was performed on the same day, and revealed an asymmetric groove along the ventrolateral portion of the balloon (figure 5). The catheter was deflated and reinflated, but without change in the contour of the balloon. The patient's treatment was deferred until reevaluation the following day. At that time, a CT scan was repeated, revealing that the asymmetric defect had resolved spontaneously (figure 6). Her brachytherapy treatment was planned using PLATO software with a fully optimized custom plan, delivering 34 Gy in 10 twice-daily fractions. As per standard procedure, she underwent daily CT imaging, which confirmed both the diameter and the symmetry of the balloon.

## Discussion

The MammoSite Catheter for APBI has shown to be well tolerated with acceptable cosmesis for the treatment of both invasive breast cancers and DCIS [4]. However, even in experienced hands, the initial MammoSite experience showed a 10% removal of implant due to asymmetry [3]. All patients described were treated using a single isotope dwell position. With a single dwell position, asymmetrical central channels would deliver an inappropriately asymmetrical dose [5]. Also, with a single dwell position, any non-spherical balloon placement would deliver an inhomogeneous dose. With a newer dose delivery technique, using dose optimization [6,7] and multiple dwell positions, some applicators forming "imperfect" spheres can be correctly treated. However, with only one channel for isotope delivery, dose optimization cannot correct for channel asymmetry within the applicator. All optimized dwell positions still deliver dose around the channel symmetrically.

After a patient has a MammoSite applicator placed percutaneously, a CT scan for planning is done very shortly thereafter. The majority of clinics can facilitate placement of the applicator, a CT scan, and planning within 4–24 hours. In case 1, our patient initially followed the typical sequence of events. In her situation, the applicator would have normally been removed, but she wished to wait and retry applicator manipulation the next day. After only 20 hours, she had intra-balloon geometry change, placing her isotope channel in the center of the balloon. Our second case was placed using the closed technique, with her initial scan following placement on the same day. Her initial scan showed an oblong applicator, which would normally be characterized as an unsuccessful placement. She was re-scanned after 4 days and without manipulation, showed a successful placement. The final patient also underwent percutaneous placement, and her initial CT was performed approximately 1–2 hours later. Again, the balloon appeared asymmetric, with marked differences in the radius of the balloon on cross-sectional imaging, which would result in inhomogeneous surface doses on a brachytherapy plan. None of these patients would have been APBI candidates as per the MammoSite study guidelines. With time, however, these patients were converted to appropriate candidates and were treated successfully.

These three patients show that asymmetry of the MammoSite applicator on an initial planning CT might not be absolute contraindications for eligibility for MammoSite-based therapy. Our institutional practice, in line with current industry standards, would consider asymmetry of 2-mm or more to be unacceptable for MammoSite treatment. These cases would have been determined to have unsuccessful placements, necessitating applicator removal as defined by the initial study guidelines. However, these patients' later scans indicated adequate symmetry without further intervention. Once the MammoSite applicator became symmetrical and spherical, the patients were treated without any difficulty. Also the applicator did not change geometry again, as evidenced by daily CT scans. Moreover, there have not, to date been instances in which the MammoSite balloon symmetry did *not *improve on repeat imaging. Although our report is admittedly limited by the small number of cases, it is nonetheless encouraging that in all instances in which balloon asymmetry was discovered, this finding soon corrected itself and remained constant thereafter.

In our experience, of approximately 75 MammoSite treatments, these three patients represent the only patients who would have been deemed poor placement due to asymmetry only. All three of these patients converted from inadequate to adequate placement over the course of 1–4 days. No factors seem to indicate this would happen, since we had all 3 patients (100%) with asymmetrical applicators convert to symmetrical applicators. Our institutional policy was to wait as long as a week, before removing the applicators. This additional week makes the total time of an indwelling MammoSite catheter to be two weeks, which is approximately the time the catheter is indwelling in the patient if placed at the time of surgery and found to be tolerable [3].

The MammoSite is still a novel technology for partial breast irradiation. As the use of MammoSite catheters increases around the country, the learning curve will continue to increase. As seen in these cases, in some unsuccessful placements of applicators due to balloon geometry, the passage of time in days has seemed to correct the geometry. This suggests that some patients previously not considered candidates for treatment could still be treated with APBI using the MammoSite, warranting further study in a prospective fashion.
